# Predictors of New‐Onset Widespread Pain in Older Adults: Results From a Population‐Based Prospective Cohort Study in the UK

**DOI:** 10.1002/art.38284

**Published:** 2014-02-25

**Authors:** John McBeth, Rosie J. Lacey, Ross Wilkie

**Affiliations:** ^1^ Arthritis Research UK Primary Care Centre Keele UniversityStaffordshireUK

## Abstract

**Objective:**

In older adults, widespread pain (WP) is common, although its etiology is unclear. This study sought to identify factors associated with an increased risk of developing WP in adults age ≥50 years.

**Methods:**

A population‐based prospective study was conducted. A baseline questionnaire was administered to subjects to collect data on pain, psychological status, lifestyle and health behaviors, and sociodemographic and clinical factors. Participants free of WP (as defined by the American College of Rheumatology 1990 criteria for fibromyalgia) were followed up for 3 years, and those with new‐onset WP at followup were identified. Logistic regression analyses were used to test the relationship between baseline factors and new‐onset WP. Multiple imputation was used to test the results for sensitivity to missing data.

**Results:**

In this population‐based study, 4,326 subjects (1,562 reporting no pain at baseline and 2,764 reporting some pain at baseline) participated at followup. Of these participants, 800 (18.5%) reported a status of new WP at followup (of whom, 121 [7.7%] had reported no pain at baseline and 679 [24.6%] had reported some pain at baseline). The majority of the study factors were associated with new‐onset WP. However, only a few factors showed a persistent association with new‐onset WP in the multivariate analysis, including age (odds ratio [OR] 0.97, 95% confidence interval [95% CI] 0.96–0.99), baseline pain status (OR 1.1, 95% CI 1.08–1.2), anxiety (OR 1.5, 95% CI 1.01–2.1), physical health‐related quality of life (OR 1.3, 95% CI 1.1–1.5), cognitive complaint (OR 1.3, 95% CI 1.04–1.6), and nonrestorative sleep (OR 1.9, 95% CI 1.2–2.8). These associations persisted after adjustment for the presence of diffuse osteoarthritis (OA), which led to a modest increase in model fit (C‐statistic 0.738, compared with 0.731 in the model excluding diffuse OA). The results were not sensitive to missing data.

**Conclusion:**

Of the factors measured in this study, nonrestorative sleep was the strongest independent predictor of new‐onset WP.

Observational studies over a 12‐month period have shown that 1 in 4 men and women age ≥65 years consult a primary care physician for evaluation of musculoskeletal pain (1). The population prevalence of musculoskeletal pain overall, independent of consultation, is higher, with between 46% and 80% of individuals age >65 years reporting experiencing pain on a daily basis (2). The prevalence of site‐specific pain in the knee, back, and shoulder typically increases with age but decreases in individuals age >65 years (4), although the prevalence of disabling pain continues to increase (5). Site‐specific pain is often attributed to osteoarthritis (OA) and allied rheumatic conditions, although it is clear that the relationship between pain reporting and underlying disease is complex (6).

In older adults, widespread pain (WP), that is, pain that affects multiple (including nonjoint) sites in the body and is the cardinal feature of fibromyalgia (7), is common, with 15% of women and 10% of men age ≥50 years reporting symptoms of WP (2), and increases with age but appears to stabilize or slightly decrease in those age ≥70 years (2). There is a strong relationship between WP and morbidity and disability in older people, including poor mental health (8) and reduced physical functioning (9). Among patients with knee OA, pain at multiple sites outside of the knee was predictive of knee pain–related disability (10) and persistent postsurgical pain after total knee replacement (11); these relationships persisted after adjustment for underlying musculoskeletal disorders, including OA, suggesting that the associations did not simply reflect the impact of underlying diffuse OA.

In working‐age adults, WP has a complex etiology, with risk factors that include physical trauma (12), although such a relationship is equivocal (13), genetic susceptibility (14), and socioeconomic status (15). In addition, psychological factors (16) are strongly associated: higher levels of psychological distress increase the risk of developing WP and, among those with WP, increase the risk of symptom persistence and associated disability (16). Lifestyle and health behaviors, such as obesity (18), poor sleep (19)_,_ smoking, and harmful alcohol use, are also associated with the onset of WP. Conversely, the absence of these common factors is predictive of musculoskeletal health in middle‐aged adults (20).

There is a paucity of available data on the etiology of WP among older adults. Health and social factors associated with poor outcomes in older adults may have a role in the onset of WP. Musculoskeletal disease (OA being the most common), comorbidity, cognitive impairment, low social networks, and restricted participation increase with age and are important predictors of physical and mental health outcomes in older adults, but their contribution to the onset of WP is unknown (21). The aim of this study was to identify factors associated with the onset of WP in older people. The study specifically sought to determine the relative contribution of socioeconomic factors, psychological distress, health‐related quality of life (HRQOL), obesity, comorbidity, and a diagnosis of OA to the onset of WP.

## PATIENTS AND METHODS

### Study design and procedure

The North Staffordshire Osteoarthritis Project (NorStOP) was a population‐based prospective cohort study conducted in the UK (2). The NorStOP sampling frame comprised all individuals age ≥50 years who were registered to receive care from 1 of 6 general practices in North Staffordshire, UK (n = 20,293). North Staffordshire is a mixed urban and rural area in the North West of England, with a population of ∼457,165 (according to the 2001 UK Census). In the UK, general practice registers offer a convenient sampling frame for population‐based studies. Since there may be duplicate registrations of individuals and since not all individuals in a community will be registered (http://www.rcgp.org.uk/pdf/ISS_INFO_02_MAY06.pdf), it is often difficult to accurately state the proportion of the UK population who are registered with a general practice. Nevertheless, it has been estimated that up to 98% of UK residents are registered in a general practice. After excluding those individuals who had recently moved or died (n = 398 [2.0%]) or were excluded from participating due to ill health (n = 77 [0.4%]), a total of 19,818 persons were eligible to participate in the current study.

### Baseline survey

All participants were mailed a baseline questionnaire that collected data on age, sex, musculoskeletal pain, psychological factors, sleep, and putative confounders. Participants were asked to provide their consent for further contact and to allow review of their medical records.

### Assessment of musculoskeletal pain

To assess musculoskeletal pain, participants were asked, “During the last month, have you had any ache or pain which has lasted for one day or longer?”. Those subjects who answered positively were asked to complete a two‐view (back and front) blank body manikin to indicate the location of their pain. These methods to determine the location and extent of pain are commonly used in population‐based studies of pain, and have been shown to be valid and reliable (22). On the basis of their reports of pain, participants were classified into 1 of 3 groups. The WP group comprised those participants who satisfied the criteria for WP included in the American College of Rheumatology 1990 criteria for fibromyalgia (24). These criteria require pain to be present above and below the waist, in the right‐ and left‐hand sides of the body, and in the axial skeleton. Those participants who reported pain that did not satisfy the criteria for WP were classified as having some pain, and those who did not report pain were classified as having no pain.

To quantify the extent of pain, participants were asked to complete the physical function scale of the Short Form 36 (SF‐36) health survey (25), which ranges from 0 to 100, with higher scores indicating better physical function. The SF‐36 has been found to be a valid measure of generic HRQOL in musculoskeletal disorders (26). For ease of interpretation, SF‐36 scores were divided by 10, and an inverse scale was used, in which increasing scores indicate poorer physical function. To capture the impact of pain on physical function, participants were further stratified as follows: those with no pain were assigned a score of 0, and those with some pain were assigned scores of 1–11, stratified by their SF‐36 physical function scores. Thus, the total baseline pain status score ranged from 0 to 11.

### Assessment of anxiety and depression

Levels of anxiety and depression were measured using the Hospital Anxiety and Depression (HAD) scale (27). The HAD scale was originally designed for use in a hospital setting but is commonly used in population‐based studies to assess the extent of an individual's symptoms of depression and anxiety. It consists of 14 items scored on a Likert scale of 0–3: 7 items address symptoms of anxiety and give a total score of 0–21, and 7 items address symptoms of depression, giving a total score of 0–21. For both scales, scores of 0–7 were classified as a noncase, scores of 8–10 were classified as a borderline case, and scores ≥11 were classified as a definite case.

### Assessment of HRQOL

The Medical Outcomes Study Short Form 12 (SF‐12) health survey is a validated shortened version of the SF‐36, an inventory originally designed to assess health status in the Medical Outcomes Study (28). The 12 questions gather information on 8 health concepts, including physical functioning, role limitations due to physical health or emotional health, mental health, bodily pain, general health, vitality, and social functioning. These items are then scored using a norm‐based method, providing a mental component summary (MCS) score and a physical component summary (PCS) score for HRQOL (28). Scores range 13–69 and 10–70 for the SF‐12 PCS and SF‐12 MCS scales, respectively, in the general US population (28). A lower score on the summary scales indicates poorer HRQOL. In the logistic regression models, for ease of interpretation, the SF‐12 PCS and MCS scores were divided by 10, and inverse scales were used, so that, in the models presented, a unit increase represents an increase in 10 scale points, with higher scores indicating poorer physical and mental HRQOL. The SF‐12 has been used in previous population‐based studies of pain (29).

Impairment of cognitive function, herein referred to as cognitive complaint, was measured using the alertness behavior subscale of the Sickness Impact Profile (30). This scale has 10 items that address the extent of alertness and ability to concentrate. Items are scored as 0 (no cognitive complaint) or 1 (cognitive complaint), with raw scores categorized to indicate no cognitive complaint (score of 0) and cognitive complaint (score >0).

### Assessment of participation

Social participation was measured using the Keele Assessment of Participation (KAP) (31). This short self‐report instrument is designed to measure, from the perspective of the individual, the extent of restriction from participation in 11 aspects of life that comprehensively measure participation in older adults. Items are phrased to capture performance (“I have”) and individual judgment, and the nature and timeliness of participation (“as and when I have wanted”). Responses are indicated on a 5‐point ordinal scale (All/Most/Some/A little/None of the time). Responders were considered restricted in an aspect of life if they did not participate in it “as and when they wanted” for “all” or “most of the time.” The resulting 11 binary items were then summed, to give a total score ranging from 0 to 11, in which increasing scores indicate more areas of restriction. The reliability and validity of the KAP have been established as adequate for providing estimates of perceived participation restriction in population studies (31).

### Assessment of social factors

Participants were asked whether they had continued their education into the level of higher education, and how they perceived the adequacy of their income (financial strain, indicated with a response of “it's a strain, need to be careful,” compared with no financial strain, indicated with a response of “can manage, comfortably well off”) (see http://surveynet.ac.uk/sqb/topics/income/qbcommentary_income_thomas.pdf). Social networks were measured using the Berkman‐Syme Social Network Index (32) (score range 0–3, categorized as high/medium network [score 2–3] or low network [score 0–1]). Area‐level employment deprivation was assessed using the Index of Multiple Deprivation (IMD) 2004 (33), specific to areas of England. The IMD is based geographically, and individuals are allocated to areas based on their postal code. These areas of employment deprivation are ranked from most to least deprived and were split using tertiles (least, middle, or most deprived).

### Assessment of lifestyle and health behaviors

The 4‐item Estimation of Sleep Problems Scale (34) was used to examine sleep quality. The scale asks about recent problems with sleep and contains items related to the most commonly occurring symptoms of poor sleep quality, including the following items: sleep onset (“During the past four weeks did you have trouble falling asleep?”), sleep maintenance (“During the past four weeks did you wake up several times per night?”), early wakening (“During the past four weeks did you have trouble staying asleep, including waking up far too early?”), and nonrestorative sleep (“During the past four weeks did you wake up after your usual amount of sleep feeling tired and worn out?”). Participants were asked to indicate the number of days in the past month in which they had experienced difficulties in each of the 4 sleep components, using a 3‐point scale ranging from 0 to 2 (0 = not at all, 1 = on some nights, 2 = on most nights). Participants were also asked to report their smoking status (current, previous, never) and alcohol use (daily, weekly, monthly, annually, never).

### Clinical factors

Body mass index (BMI) was calculated from the participants' reported height and weight. Based on these calculations, participants were classified as either underweight (BMI <20 kg/m^2^), normal range (20–24.99 kg/m^2^), overweight (≥25–29.99 kg/m^2^), or obese (≥30 kg/m^2^).

Comorbidity was defined using 2 methods: self‐report of health conditions and impairments, and general practice consultation data. Self‐report data were used because these types of data reflect an individual's perception of how he or she appraises the presence of morbidities and how he or she may relate to the use of health and social care. Participants were asked to report the presence of 3 common chronic health conditions in older adults (chest problems, heart problems, diabetes) and 6 impairments most commonly associated with disability (deafness, problems with eyesight, cough with spit, breathless when walking, dizziness, weakness in arms/legs). From these single items, counts of health conditions (range 0–3) and impairments (range 0–6) were calculated.

General practice consultation data were used to provide an objective assessment of morbidities, for comparison. General practitioners in the study populations used the Read system to code all morbidities during actual consultations. Read codes are used in primary care by general practitioners in the UK to record morbidity data on clinical computer systems (35). The Read codes cross‐map to International Classification of Diseases, Ninth Revision (ICD‐9)/ICD‐10 codes (for diseases). Morbidity data (i.e., symptoms and diseases) in this system are grouped under 19 main Read chapters. Data collected at the second hierarchical level or above were used to identify morbidity, and were related to at least one consultation for a given morbidity category in the 18 months prior to baseline (repeat consultations for the same morbidity were not included). A simple count of the number of morbidities identified in a consultation was calculated.

To define diffuse OA, we first identified those participants for whom a potential diagnosis of OA was recorded as the reason for consultation in the general practice notes (code N05). Subjects with a recorded consultation for OA were asked about the presence of pain in the 4 most prevalent areas of OA (hands, hips, knees, and feet) in older adults (36). This classification satisfies the criteria for the clinical syndrome of OA (36). Individuals were categorized as having diffuse OA (consultation for OA and reported pain in at least 2 areas), single‐site OA (consultation for OA and reported pain in 1 area), or no OA (no consultation for OA).

### Followup survey

Participants who returned the baseline questionnaire, who were free of WP at baseline, and who agreed to further contact were mailed a followup questionnaire 36 months later. To assess the presence of pain at followup, methods identical to those used in the baseline survey were utilized. Participants who reported having WP at followup were classified as having new‐onset WP, and those who reported having no WP at followup were classified as being WP‐free.

### Ethics approval

Ethics approval was obtained from the North Staffordshire Local Research Ethics Committee. Consent was implied through the returned questionnaire, and participants provided additional consent to medical record review.

### Statistical analysis

First, the distribution of demographic, psychological, social and lifestyle factors, and health behaviors and clinical factors was examined in the participants according to pain status at followup (new‐onset WP versus WP‐free), with differences tested for significance using chi‐square or Kruskal‐Wallis tests, as appropriate. Second, a complete‐case analysis was conducted, which included only those participants who provided full questionnaire data. Univariate logistic regression models were constructed to examine the relationship between baseline factors and the onset of WP at followup, adjusting for age and sex. A multivariate model was constructed that included all factors except diffuse OA. In a final multivariate model, diffuse OA was included. To evaluate model fit, the concordance indexes (C‐statistic) were calculated. A C‐statistic of 0.50 indicates the predictive ability of a model to be no better than chance, 0.7 indicates reasonable predictive ability, 0.8 indicates high predictive ability, and 1.0 indicates perfect predictive ability (37). To determine whether any associations were moderated by age, an interaction term between age (included as a binary variable based on the mean age, i.e., 50–64 years compared to ≥65 years) and each baseline variable was examined in univariate analysis. Similarly, to determine whether the associations between baseline factors and new‐onset WP were moderated by nonconsent to medical record review, an interaction term was examined in univariate analysis. Significant interactions were included in the final multivariate models. Sensitivity to missing data was examined via multiple imputation analysis (38) (for rationale and details of the sensitivity analysis, see Supplementary Appendix 1, available online at the *Arthritis & Rheumatology* web site at http://onlinelibrary.wiley.com/doi/10.1002/art.38284/abstract). Since the complete‐case and imputed analyses produced the same results, the data for the complete‐case analysis are presented.

Stata statistical software release 11 (StataCorp) was used for all analyses. The results of the analyses are presented as odds ratios (ORs) with 95% confidence intervals (95% CIs). For all analyses, the WP‐free group was classified as the referent category.

## RESULTS

**Figure 1:**
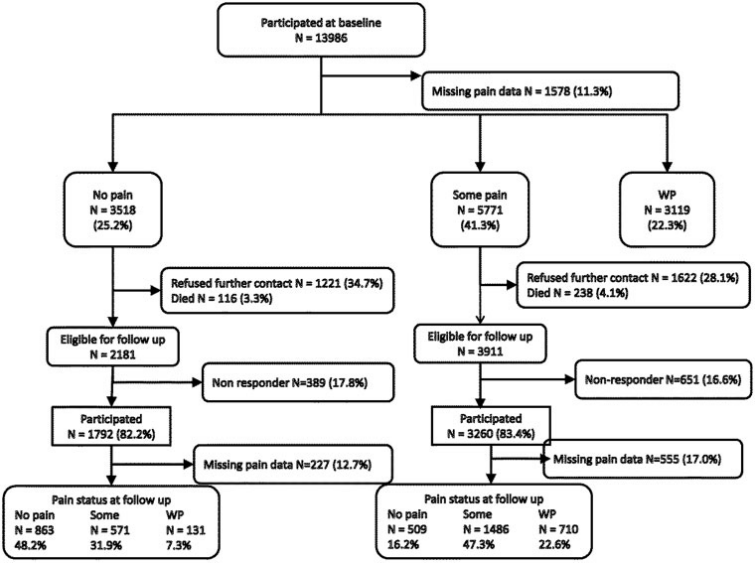
Distribution of the study participants at baseline and 36 months of followup. WP = widespread pain.

Of the 19,818 participants who were eligible to take part in the study, 13,986 (70.6%) returned a completed questionnaire. Of those, 12,408 (88.7%) provided data on pain status and on the various study factors (Figure 1). Compared to participants, nonparticipants were younger (median age 71 years versus 65 years) and more likely to be male (10.7% versus 11.8%). A total of 3,119 participants (22.3%) reported having WP, 3,518 (25.2%) were pain‐free, and 5,771 (41.3%) reported having some pain at baseline. After excluding those with WP at baseline, those who refused further contact, decedents, and nonresponders to the followup questionnaire, 1,791 participants with no pain at baseline and 3,203 with some pain at baseline were available for analysis at followup, and of those, 229 (12.8%) and 439 (13.7%), respectively, did not provide data on pain status at followup, leaving a total of 4,326 participants (of whom, 1,562 had reported no pain at baseline and 2,764 had reported some pain at baseline) in the final analysis.

After 36 months of followup, 800 participants (18.5%) reported having new‐onset WP, and 3,526 (81.5%) reported being WP‐free (Table 1). There was no difference in the median age between the new‐onset WP and WP‐free groups at followup. Women were more likely than men to report new onset of WP at followup. A total of 24.6% of the participants who had reported some pain at baseline (n = 679) developed new‐onset WP, compared to 7.7% of those who were pain‐free at baseline (n = 121) (*P* < 0.0001). The prevalence of new‐onset WP was highest in participants who had not continued into higher education, those who were outside the normal range of BMI, those who lived in the areas of middle and most deprived employment deprivation, those who reported financial strain, and those who reported restricted social participation. The rate of new‐onset WP at followup was higher among participants who were classified as having a possible or probable case of anxiety or depression, and among those with lower physical and mental HRQOL (*P* < 0.0001 for all comparisons). In addition, new‐onset WP at followup was associated with a greater number of health conditions and health impairments at baseline, as well as with more comorbidities at baseline.

**Table 1:** Characteristics of the study participants at baseline*

	No. available for analysis	WP‐free (n = 3,526)	New‐onset WP (n = 800)	*P*
Age, median (IQR) years	4,326	63 (56–71)	63 (56–70)	0.74
Sex				
Male	2,046	1,710 (83.6)	336 (16.4)	<0.001
Female	2,280	1,816 (79.7)	464 (20.4)	
Baseline pain status score, median (IQR) (range 0–11)	4,209	1.5 (0–3.5)	4 (2–7)	<0.001
Higher education				
No	3,635	2,950 (81.2)	685 (18.8)	0.04
Yes	618	522 (84.5)	96 (15.5)	
Area of employment deprivation				
Least	1,669	1,408 (84.4)	261 (15.6)	<0.001
Middle	1,480	1,186 (80.1)	294 (19.9)	
Most	1,175	931 (79.2)	244 (20.8)	
Financial strain				
No	2,680	2,256 (84.2)	424 (15.8)	<0.001
Yes	1,596	1,236 (77.4)	360 (22.6)	
Social networks				
High	1,360	1,118 (82.2)	242 (17.8)	0.29
Low	2,138	1,727 (80.8)	411 (19.2)	
Smoking				
Never	1,869	1,546 (82.7)	323 (17.3)	0.04
Previous	1,842	1,499 (81.4)	343 (18.6)	
Current	583	455 (78.1)	128 (22.0)	
Alcohol use				
Daily	983	835 (84.9)	148 (15.1)	0.003
1–2 per week	1,619	1,322 (81.7)	297 (18.3)	
1–2 per month	673	541 (80.4)	132 (19.6)	
1–2 per year	601	487 (81.0)	114 (19.0)	
Never	408	311 (76.2)	97 (23.8)	
Social participation score, median (IQR) (range 0–11)	4,326	0 (0–1)	0 (0–2)	<0.001
Anxiety				
None	2,919	2,488 (85.2)	431 (14.8)	<0.001
Borderline	791	603 (76.2)	188 (23.8)	
Definite	616	435 (70.6)	181 (29.4)	
Depression				
None	3,729	3,095 (83.0)	634 (17.0)	<0.001
Borderline	346	245 (70.8)	101 (29.2)	
Definite	251	186 (74.1)	65 (25.9)	
SF‐12 score, median (IQR) (range 0–10)				
MCS	3,950	4.46 (4.28–5.26)	4.74 (4.22–6.03)	<0.001
PCS	3,950	4.96 (4.46–6.19)	6.09 (4.97–7.04)	<0.001
Cognitive complaint				
No	2,654	2,272 (85.6)	382 (14.4)	<0.001
Yes	1,672	1,254 (75.0)	418 (25.0)	
Health conditions				
None	3,002	2,494 (83.1)	508 (16.9)	<0.001
1	1,077	853 (79.2)	224 (20.8)	
≥2	247	179 (72.5)	68 (27.5)	
Health impairments				
None	1,594	1,380 (86.6)	214 (13.4)	<0.001
1	1,473	1,228 (83.4)	245 (16.7)	
≥2	1,259	918 (72.9)	341 (27.1)	
Comorbidities score, median (IQR) (range 0–18)	3,247	2 (1–4)	3 (1–5)	<0.001
BMI				
Normal range	1,541	1,302 (84.5)	239 (15.5)	<0.001
Underweight	121	100 (82.6)	21 (17.4)	
Overweight	1,832	1,483 (81.0)	349 (19.1)	
Obese	705	538 (76.3)	167 (23.7)	
OA				
None	2,452	2,108 (86.0)	344 (14.0)	<0.001
Single site	503	426 (84.7)	77 (15.3)	
Diffuse	1,030	713 (69.2)	317 (30.8)	
Trouble with sleep onset				
No	1,898	1,622 (85.5)	276 (14.5)	<0.001
Some nights	1,936	1,541 (79.6)	395 (20.4)	
Most nights	415	303 (73.0)	112 (27.0)	
Trouble with sleep maintenance				
No	863	760 (88.1)	103 (11.9)	<0.001
Some nights	2,298	1,889 (82.2)	409 (17.8)	
Most nights	1,080	809 (74.9)	271 (25.1)	
Trouble staying asleep				
No	1,660	1,398 (87.4)	202 (12.6)	<0.001
Some nights	1,927	1,539 (79.9)	388 (20.1)	
Most nights	662	474 (71.6)	188 (28.4)	
Nonrestorative sleep				
No	1,897	1,663 (87.7)	234 (12.3)	<0.001
Some nights	1,863	1,485 (79.7)	378 (20.3)	
Most nights	477	306 (64.2)	306 (35.9)	

Participants with missing data are excluded. Except where indicated otherwise, values are the number (%) of participants. WP = widespread pain; IQR = interquartile range; SF‐12 = Short Form 12; MCS = mental component summary; PCS = physical component summary; BMI = body mass index; OA = osteoarthritis.

Furthermore, having a diagnosis of diffuse OA was also associated with new‐onset WP. At followup, 14.1% of those with no recorded OA diagnosis developed WP, compared to 15.3% of those who had single‐site OA and 30.8% of those who had diffuse OA (*P* < 0.0001).

Table 2 shows the results of the logistic regression analyses. After adjusting for age and sex, all factors, with the exception of social networks, were associated with new‐onset WP at followup. When these factors were entered into a multivariate model, age (OR 0.97, 95% CI 0.96–0.99), baseline pain status score (OR 1.1, 95% CI 1.08–1.2), definite anxiety (OR 1.5, 95% CI 1.01–2.1), SF‐12 PCS score indicating worse physical HRQOL (OR 1.3, 95% CI 1.1–1.5), presence of cognitive complaint (OR 1.3, 95% CI 1.04–1.6), and nonrestorative sleep on most nights (OR 1.9, 95% CI 1.2–2.8) were associated with reporting new‐onset WP at followup.

**Table 2:** Predictors of new‐onset widespread pain*

Exposure	Univariate model, OR (95% CI)^a^	Multivariate model	
		Excluding OA, OR (95% CI)	Including OA, OR (95% CI)
Age (in years)	1.0 (0.99–1.0)	0.97 (0.96–0.99)	0.97 (0.96–0.99)
Sex			
Male	Referent	Referent	Referent
Female	1.3 (1.1–1.5)	1.1 (0.9–1.4)	1.1 (0.9–1.4)
Baseline pain status score (range 0–11)	1.2 (1.2–1.2)	1.1 (1.08–1.2)	1.2 (1.1–1.3)
Higher education			
No	Referent	Referent	Referent
Yes	1.3 (1.03–1.7)	1.1 (0.8–1.5)	1.1 (0.8–1.6)
Area of employment deprivation			
Least	Referent	Referent	Referent
Middle	1.3 (1.1–1.6)	1.2 (0.9–1.5)	1.2 (0.9–1.5)
Most	1.4 (1.2–1.7)	1.0 (0.7–1.3)	0.98 (0.7–1.3)
Financial strain			
No	Referent	Referent	Referent
Yes	1.6 (1.3–1.8)	1.1 (0.9–1.4)	1.1 (0.9–1.4)
Social networks			
High	Referent	Referent	Referent
Low	1.1 (0.9–1.3)	0.9 (0.7–1.1)	0.9 (0.7–1.1)
Smoking			
Never	Referent	Referent	Referent
Previous/current	1.2 (1.1–1.3)	1.1 (0.95–1.3)	1.1 (0.95–1.3)
Alcohol use			
Daily	Referent	Referent	Referent
Infrequent/never	1.1 (1.03–1.2)	1.0 (0.95–1.1)	1.0 (0.95–1.1)
Social participation score (range 0–11)	1.2 (1.2–1.3)	0.9 (0.9–1.0)	0.9 (0.9–1.0)
Anxiety			
None	Referent	Referent	Referent
Borderline	1.8 (1.5–2.1)	1.2 (0.9–1.6)	1.2 (0.9–1.6)
Definite	2.3 (1.9–2.9)	1.5 (1.01–2.1)	1.4 (1.0–2.1)
Depression			
None	Referent	Referent	Referent
Possible	2.0 (1.6–2.6)	1.0 (0.6–1.4)	1.0 (0.7–1.5)
Probable	1.7 (1.3–2.3)	0.7 (0.4–1.2)	0.7 (0.4–1.2)
SF‐12 score (range 0–10)			
MCS	1.3 (1.2–1.4)	1.0 (0.9–1.2)	1.0 (0.9–1.2)
PCS	1.7 (1.6–1.9)	1.3 (1.1–1.5)	1.2 (1.1–1.5)
Cognitive complaint			
No	Referent	Referent	Referent
Yes	2.0 (1.7–2.3)	1.3 (1.04–1.6)	1.3 (1.0–1.6)
BMI			
Normal	Referent	Referent	Referent
Underweight	1.1 (0.7–1.8)	1.5 (0.7–2.9)	1.5 (0.8–3.0)
Overweight	1.3 (1.1–1.6)	0.9 (0.7–1.2)	0.9 (0.7–1.1)
Obese	1.7 (1.4–2.1)	0.9 (0.6–1.2)	0.8 (0.6–1.1)
Health conditions			
None	Referent	Referent	Referent
1	1.4 (1.1–1.6)	0.8 (0.6–1.1)	0.9 (0.7–1.1)
2–3	2.0 (1.5–2.7)	0.9 (0.6–1.5)	−0.9 (0.6–1.5)
Health impairments			
None	Referent	Referent	Referent
1	1.4 (1.1–1.6)	0.9 (0.7–1.2)	0.97 (0.6–1.5)
2–6	2.6 (2.1–3.1)	1.2 (0.9–1.6)	1.2 (0.9–1.6)
Comorbidities score (range 0–18)	1.1 (1.06–1.1)	1.0 (0.9–1.0)	0.97 (0.9–1.0)
OA			
None	Referent	–	Referent
Single site	1.1 (0.9–1.5)	–	0.98 (0.7–1.4)
Diffuse	2.7 (2.3–3.3)	–	1.7 (1.3–2.1)
Trouble with sleep onset			
No	Referent	Referent	Referent
Some nights	1.5 (1.2–1.7)	11.1 (0.8–1.4)	1.1 (0.8–1.4)
Most nights	2.1 (1.6–2.7)	0.9 (0.5–1.3)	0.8 (0.5–1.3)
Trouble with sleep maintenance			
No	Referent	Referent	Referent
Some nights	11.6 (1.2–2.0)	11.2 (0.9–1.6)	1.2 (0.9–1.8)
Most nights	2.4 (1.9–3.1)	1.1 (0.7–1.7)	1.1 (0.7–1.7)
Trouble staying asleep			
No	Referent	Referent	Referent
Some nights	1.7 (1.4–2.0)	1.2 (0.9–1.6)	1.2 (0.8–1.6)
Most nights	2.7 (2.1–3.3)	1.2 (0.8–1.9)	1.2 (0.8–2.0)
Nonrestorative sleep			
No	Referent	Referent	Referent
Some nights	1.8 (1.5–2.1)	1.3 (0.99–1.7)	1.3 (0.98–1.7)
Most nights	3.9 (3.1–4.9)	1.9 (1.2–2.8)	1.8 (1.2–2.8)
C‐statistic		0.731	0.738

OA = osteoarthritis; OR = odds ratio; 95% CI = 95% confidence interval; SF‐12 = Short Form 12; MCS = mental component summary; PCS = physical component summary; BMI = body mass index.

a Model adjusted for age, sex, or age and sex, as appropriate.

In the final multivariate model, diffuse OA was included as a covariate. Although a diagnosis of diffuse OA was significantly associated with having new‐onset WP at followup (OR 1.7, 95% CI 1.3–2.1), the associations with age, baseline pain status score, SF‐12 PCS score, cognitive complaint, and nonrestorative sleep on most nights persisted and were not attenuated by the addition of diffuse OA as a factor. The model fit increased modestly with the addition of diffuse OA (model C‐statistic 0.738, compared to 0.731 in the model excluding diffuse OA). The relationship between baseline variables and new‐onset WP was not moderated by age (*P* > 0.05). There was a significant, less‐than‐multiplicative interaction between nonconsent to medical record review, lack of continuation into higher education, and new‐onset WP (OR 0.3, 95% CI 0.2–0.7). Inclusion of this interaction term did not affect the associations in the multivariate models.

## DISCUSSION

This study aimed to identify the determinants of developing new‐onset WP in a cohort of community‐dwelling older people. At baseline, reporting musculoskeletal pain was common, with just under one‐half of the cohort reporting having some pain and one‐quarter reporting having WP. Of those participants who were free of WP at baseline, 19% reported new onset of WP at followup. The rate of new‐onset WP was higher in those reporting some pain at baseline (24.6%) when compared to those who were pain free at baseline (7.7%). Although the majority of factors measured at baseline were associated with new‐onset WP, relatively few of the factors were independently associated in multivariate analyses. When diffuse OA was excluded from the model, the independent predictors of new‐onset WP at followup were age, baseline pain status score, anxiety, poorer physical HRQOL, presence of cognitive complaint, and nonrestorative sleep on most nights. The C‐statistic for model fit (0.731) suggested that the ability of this model to predict new‐onset WP was reasonable (37). Even though a diagnosis of diffuse OA was independently associated with new‐onset WP in the multivariate model, the addition of this factor was associated with only a very modest increase in model fit (C‐statistic 0.738).

The study has several strengths. It was a large, population‐based study of an unselected population. The response rate was high and was comparable to that in other population‐based studies that have used postal questionnaires. The sensitivity analysis indicated that questionnaire nonresponse and missing data did not introduce nonresponse bias to the results. There was an interaction between nonconsent to medical record review and lack of further education in association with new‐onset WP; however, this did not impact the results in the multivariate models.

The available data covered a number of important areas in relation to developing WP and, specifically, factors that may influence pain reporting in older people. One limitation is that the data were self‐reported. While questionnaires such as the HAD have been validated for use in general population samples and in postal surveys, the validity and reliability of the other measures used in the present study are less clear. For example, use of self‐reported height and weight to classify BMI among older adults has been associated with misclassification and underestimation of rates of underweight and obese persons (39), and these effects were particularly marked among the oldest subjects. However, in another study, the data were adjusted for sociodemographic factors, including sex, marital status, and household income, which resulted in a close approximation of actual BMI (40).

More important in this study was the method used to assess the presence of pain. Pain was assessed using blank body manikins, a standard data‐capture method used in postal surveys. High levels of interrater reliability for pain scoring (κ > 0.60) and subsequent classification of WP (κ = 0.98) have been demonstrated using this data‐capture method (23). However, while this method has been shown to be a valid and reliable tool for the assessment of pain in mid‐life adults, the validity of measuring the presence and extent “of” pain using body manikins in older people is less clear. For example, which pains do older people report? All pain? The most bothersome? Or the most intense? Rates of agreement between manikin‐derived pain measures and written responses to direct questions regarding site‐specific disorders, such as low back pain and neck pain, have been found to be high (75% across all disorders), but rates of agreement decreased with age (41). However, in the latter study, older people did not systematically underreport site‐specific pain on the body manikin, and there are no data available for WP.

In the multivariate analyses, increasing age was associated with a decreased likelihood of reporting new‐onset WP. This is an interesting observation, since, at age 50 years and older, age‐related rates of chronic disease start to rise dramatically, including rates of OA. General practitioners' labeling of joint pain starts to reflect the probability that the reason for joint pain is likely to be OA from this age upward. However, not all people with OA will consult a general practictioner, and adjusting for age will capture those individuals. We could hypothesize that if OA were associated with new‐onset WP, and since the prevalence of OA increases with age, the incidence of WP would increase with age.

In addition, theoretically, WP should increase with age simply because of the continuing opportunity to accumulate pain over time, particularly through age‐related musculoskeletal diseases. Instead, the results of this and previous studies suggest that, in the general population, WP decreases in the oldest individuals (2). The prevalence of fibromyalgia, a disorder characterized by WP in the presence of multiple other somatic symptoms, including fatigue, anxiety, and depression, increases with age up until about the sixth decade of life, and decreases thereafter, in population samples in the US (42), Canada (43), Spain (29), Finland (44), and Brazil (45). The results of 2 studies have suggested that the prevalence of fibromyalgia continues to increase in older people beyond the sixth decade, with prevalence peaking among US adults age 70–79 years (46) and among individuals from 5 European countries (France, Germany, Italy, Portugal, and Spain) age 75–84 years (47).

Irrespective of the age at which prevalence peaks, all of these studies demonstrate that the prevalence of WP and/or fibromyalgia decreases in the oldest individuals in a population. There are a number of reasons why the prevalence of WP may decline with age. Changes in exposures over time will influence the reporting of WP, one obvious example being the absence, in the oldest individuals, of occupational exposures that are strongly associated with WP (16). Furthermore, older people may underreport pain because of social factors, perceptions that pain is a natural part of aging, or stoicism (47).

The final multivariate models indicated that sociodemographic, psychological, and clinical factors were reasonable predictors of new‐onset WP in older adults. Although the presence of diffuse OA (diagnosed in the 2 years prior to the baseline survey) was also a predictor of the development of WP, inclusion of this exposure, which is commonly considered to be the most common cause of musculoskeletal pain in older adults, in the final model was associated with only a modest increase in the model fit. The C‐statistic indicates that the prediction of new cases of WP could be improved with the inclusion of additional factors, along with the factors already assessed in the present study.

Currently, the management and treatment of musculoskeletal pain in older adults is suboptimal (48). In this study, nonrestorative sleep was the strongest predictor of new‐onset WP. We have previously shown that among individuals with WP, restorative sleep was a predictor of symptom resolution (19). Taken together, these data suggest that sleep may offer a modifiable target to improve outcomes in this patient group. The clinical effectiveness of pharmacologic and nonpharmacologic approaches to sleep should be tested in randomized controlled trials.

Anxiety is another potentially modifiable factor that was identified in the present study and that is amenable to intervention. Future studies should seek to identify other factors that increase the risk of developing new‐onset WP in older people and that may offer targets for pain reduction. Such mechanisms are likely to be multifactorial, including common factors known to be associated with reporting WP, age‐specific social factors that influence pain reporting, and changes in pain‐processing mechanisms. In studies of working‐age adults, we have previously shown that psychosocial factors were strong predictors of new‐onset WP (17), and that the risks conferred by those factors were moderated by altered functioning of stress‐response systems, including the hypothalamic–pituitary–adrenal stress axis (49). However, whether these processes are important in older adults is not known. Recent reports have suggested that similar psychosocial risk factors are as common among older as among younger people, and these factors may be predictive of poor quality of life among individuals age ≥65 years independently of physical and mental illness (50). The role of these psychosocial factors and new‐onset WP in older people may be a useful line of future inquiry.

In conclusion, this study shows that new‐onset WP is common in older adults and can be predicted on the basis of a number of factors. A diagnosis of diffuse OA was associated with new‐onset WP, indicating that underlying disease may contribute to the onset of multi‐site pain. Clinical approaches that target multiple sites of OA involvement may be useful. However, the clinical approach to managing WP in older adults may need to move beyond focusing on treatment of OA alone and might consider combined interventions. This study suggests that in addition to OA, sleep, cognitive impairment, anxiety, and physical health may be important treatment targets. Further research to identify other factors that are predictive of WP and are potential treatment targets in older adults is also indicated. These studies should explore age‐specific factors, including social factors and pain processing, and psychosocial mechanisms that are robust predictors of new‐onset WP in working‐age adults.

## AUTHOR CONTRIBUTIONS

All authors were involved in drafting the article or revising it critically for important intellectual content, and all authors approved the final version to be published. Dr. McBeth had full access to all of the data in the study and takes responsibility for the integrity of the data and the accuracy of the data analysis.

**Study conception and design.** McBeth, Lacey, Wilkie.

**Acquisition of data.** McBeth, Lacey, Wilkie.

**Analysis and interpretation of data.** McBeth, Lacey, Wilkie.

## Supplementary Material

Additional Supporting Information may be found in the online version of this article.

Supplementary Data
