# Supplementary material for: Predictors of New‐Onset Widespread Pain in Older Adults: Results From a Population‐Based Prospective Cohort Study in the UK
Source: Arthritis Rheumatol. 2014 Feb 25;66(3):757–67. doi: 10.1002/art.38284 (PMC4163719; doi:10.1002/art.38284)
Supplement: Supplementary file 1 — Supplementary Data [file art-66-757-s2.doc]

**Supplementary Appendix 1 for McBeth et al**

Complete-case analysis results in a smaller dataset and a consequent loss of statistical power ^1^ but more importantly may introduce bias to the results ^2^. We were concerned to test the sensitivity of the study results to missing data and in a second phase of analysis used multiple imputation (MI) to impute the missing values in the data set. MI is a statistical technique that uses all observed data to impute plausible values for the missing values. The imputed values are not real values and have no interpretation ^3^ but rather are statistical tools that enable the non-missing variables from a particular subject to be used in the analysis. In MI the missing values are imputed *M* times resulting in a range of values for the missing value and an estimate of variance that reflects the uncertainty around the imputed value. For the current analyses *M* = 20 ^4^. The *M* datasets are analysed separately and the results are combined to give a summary statistic. MI has been shown to be superior to complete case analyses and other methods of data imputation such as the missing indicator method ^1,5^. In the current analysis missing values were assumed to be missing at random (MAR), i.e. that the missing values were related to other, observed, variables and could be predicted by the values of those observed variables ^6^. MI was based on the procedure described by Raghunathan et al ^7^ that imputes continuous variables using linear regression and categorical variables using polytomous regression. In the MI analysis unordered multinomial logistic regression was used to examine the relationships between age, pain status and putative explanatory variables. The STATA *ice* command was used to generate the imputed datasets and the *mi* procedure used to analyse the imputed datasets.

Appendix 1 references

1. Janssen KJ, Donders AR, Harrell FE Jr, Vergouwe Y, Chen Q, Grobbee DE, Moons KG. Missing covariate data in medical research: to impute is better than to ignore. J Clin Epidemiol. 2010 Jul;63(7):721-7.
2. Knol MJ, Janssen KJ, Donders AR, Egberts AC, Heerdink ER, Grobbee DE, Moons KG, Geerlings MI. Unpredictable bias when using the missing indicator method or complete case analysis for missing confounder values: an empirical example. J Clin Epidemiol. 2010 Jul;63(7):728-36.
3. Taylor JM, Cooper KL, Wei JT, Sarma AV, Raghunathan TE, Heeringa SG. Use of multiple imputation to correct for nonresponse bias in a survey of urologic symptoms among African-American men. Am J Epidemiol. 2002 Oct 15;156(8):774-82.
4. Graham JW, Olchowski AE, Gilreath TD. How many imputations are really needed? Some practical clarifications of multiple imputation theory. Prev Sci. 2007 Sep;8(3):206-13.
5. van der Heijden GJ, Donders AR, Stijnen T, Moons KG. Imputation of missing values is superior to complete case analysis and the missing-indicator method in multivariable diagnostic research: a clinical example. J Clin Epidemiol. 2006 Oct;59(10):1102-9.
6. Horton NJ, Kleinman KP. Much ado about nothing: A comparison of missing data methods and software to fit incomplete data regression models. Am Stat. 2007 Feb;61(1):79-90.
7. Raghunathan T.E., Lepkowski J.M., VanHoewyk J., Solenberger P., (2001). A multivariate technique for multiply imputing missing values using a sequence of regression models. Survey Methodology, 27, 85-95.
